# School for Health Officers, Conducted by Harvard University and the Massachusetts Institute of Technology

**Published:** 1913-09

**Authors:** 


### School for Health Officers, Conducted by Harvard University and the Massachusetts Institute of Technology.

Beginning this fall Harvard University and the Massachusetts Institute of Technology are to maintain in coöperation a School for Public Health Officers. The facilities of both institutions are to be available to students in the School and the Certificate of Public Health (C. P. H.) is to be signed by both President Lowell and President Maclaurin.

The object of this school is to prepare young men for public health work, especially, to fit them to occupy administrative and executive positions such as health officers or members of boards of health, as well as secretaries, agents, and inspectors of health organizations.

It is recognized that the requirements for public health service are broad and complicated, and that the country needs leaders in every community, fitted to guide and instruct the people on all questions relating to the public health. To this end, the instruction of the new school will be on the broadest lines. It will be given by lectures, laboratory work, and other forms of instruction offered by both institutions, and also by special instructors from national, state, and local health agencies.

The requirements for admission are such that graduates of colleges, or technical and scientific schools, who have received adequate instruction in physics, chemistry, biology, and French or German, may be admitted to the school. The medical degree is not in any way prerequisite for admission, although the administrative board strongly urges men who intend to specialize in public health work to take the degree of M. D. before they become members of the School for Health Officers.

The administrative board which will conduct the new school is composed of Professor William T. Sedgwick, of the Massachusetts Institute of Technology; Professor Milton J. Rosenau, of Harvard; and Professor George C. Whipple, of Harvard. Professor Rosenau, of Harvard, has the title of Director, and the work of the school will be under his immediate supervision.

